# Supplementary material for: Population genetic structure of Texas horned lizards: implications for reintroduction and captive breeding
Source: PeerJ. 2019 Oct 1;7:e7746. doi: 10.7717/peerj.7746 (PMC6777493; doi:10.7717/peerj.7746)
Supplement: Table S1 — N is the number of individuals; NA is the number of alleles; HO is observed heterozygosity; HE is expected heterozygosity; FIS is the inbreeding coefficient; Null is the frequency of null alleles (Brookfield, 1996 eq1), * indicates evidence for null alleles as determined by MICRO-CHECKER and **—indicates significant heterozygote deficit after sequential Bonferroni correction as determined by GENEPOP (α = 0.05). For sampling site names, SP = state park, WMA = wildlife management area, CMA = Cross Bar Management Area, Co. = county, RPQRR = Rolling Plains Quail Research Ranch. [file peerj-07-7746-s001.docx]

| Site | Locus | **N** | **N_A_** | **H_O_** | **H_E_** | **F_IS_** | **Null** |
| --- | --- | --- | --- | --- | --- | --- | --- |
| Brewster Co. | *Pc41* | 31 | 16 | 0.903 | 0.912 | 0.010 | 0.005 |
|  | *PcD01* | 30 | 23 | 0.933 | 0.915 | -0.020 | -0.010 |
|  | *PcD09* | 31 | 11 | 0.710 | 0.805 | 0.119 | 0.053 |
|  | *Pc70* | 31 | 7 | 0.677 | 0.786 | 0.138 | 0.061 |
|  | *PcD14* | 31 | 12 | 0.935 | 0.891 | -0.050 | -0.023 |
|  | *PcD20* | 31 | 16 | 0.774 | 0.900 | 0.140 | **0.066*** |
|  | *PcD52* | 31 | 8 | 0.548 | 0.774 | 0.291 | **0.127*** |
|  | *Pc83* | 31 | 12 | 0.677 | 0.644 | -0.052 | -0.020 |
|  | *PcD26* | 31 | 10 | 0.903 | 0.860 | -0.050 | -0.023 |
| . | *PcD53* | 31 | 15 | 0.839 | 0.840 | 0.002 | 0.0008 |
| Hueco Tanks SP | *Pc41* | 12 | 11 | 0.833 | 0.885 | 0.059 | 0.028 |
|  | *PcD01* | 12 | 11 | 0.917 | 0.872 | -0.052 | -0.024 |
|  | *PcD09* | 12 | 6 | 0.667 | 0.642 | -0.038 | -0.015 |
|  | *Pc70* | 12 | 8 | 0.833 | 0.854 | 0.024 | 0.011 |
|  | *PcD14* | 12 | 8 | 0.833 | 0.809 | -0.030 | -0.013 |
|  | *PcD20* | 12 | 9 | 0.833 | 0.767 | -0.086 | -0.037 |
|  | *PcD52* | 12 | 2 | 0.417 | 0.469 | 0.111 | 0.036 |
|  | *Pc83* | 12 | 9 | 0.750 | 0.847 | 0.115 | 0.053 |
|  | *PcD26* | 12 | 8 | 0.833 | 0.809 | -0.030 | -0.013 |
|  | *PcD53* | 12 | 10 | 1.000 | 0.806 | -0.241 | -0.108 |
| Seminole Canyon SP | *Pc41* | 17 | 13 | 0.941 | 0.889 | -0.058 | -0.028 |
|  | *PcD01* | 17 | 12 | 0.941 | 0.898 | -0.048 | -0.023 |
|  | *PcD09* | 17 | 11 | 0.882 | 0.827 | -0.067 | -0.030 |
|  | *Pc70* | 17 | 8 | 0.706 | 0.820 | 0.139 | 0.063 |
|  | *PcD14* | 17 | 15 | 0.824 | 0.917 | 0.102 | 0.049 |
|  | *PcD20* | 17 | 12 | 0.941 | 0.888 | -0.060 | -0.028 |
|  | *PcD52* | 17 | 7 | 0.941 | 0.803 | -0.172 | -0.077 |
|  | *Pc83* | 16 | 14 | 0.938 | 0.900 | -0.041 | -0.020 |
|  | *PcD26* | 17 | 9 | 0.882 | 0.858 | -0.028 | -0.013 |
|  | *PcD53* | 17 | 10 | 0.647 | 0.633 | -0.022 | -0.009 |
| Midland Co. | *Pc41* | 30 | 11 | 0.867 | 0.857 | -0.012 | -0.005 |
|  | *PcD01* | 30 | 14 | 0.900 | 0.868 | -0.036 | -0.017 |
|  | *PcD09* | 30 | 11 | 0.867 | 0.886 | 0.022 | 0.010 |
|  | *Pc70* | 30 | 9 | 0.833 | 0.809 | -0.030 | -0.013 |
|  | *PcD14* | 30 | 14 | 0.833 | 0.884 | 0.058 | 0.027 |
|  | *PcD20* | 30 | 28 | 0.967 | 0.943 | -0.025 | -0.012 |
|  | *PcD52* | 30 | 8 | 0.767 | 0.817 | 0.061 | 0.028 |
|  | *Pc83* | 30 | 17 | 0.767 | 0.834 | 0.081 | 0.037 |
|  | *PcD26* | 30 | 10 | 0.833 | 0.868 | 0.040 | 0.018 |
|  | *PcD53* | 30 | 10 | 0.633 | 0.626 | -0.012 | -0.005 |
| Yoakum Dunes WMA | *Pc41* | 36 | 12 | 0.861 | 0.858 | -0.004 | -0.002 |
|  | *PcD01* | 36 | 15 | 0.917 | 0.894 | -0.025 | -0.012 |
|  | *PcD09* | 36 | 12 | 0.917 | 0.864 | -0.061 | -0.028 |
|  | *Pc70* | 36 | 9 | 0.833 | 0.840 | 0.008 | 0.004 |
|  | *PcD14* | 36 | 12 | 0.917 | 0.883 | -0.038 | -0.018 |
|  | *PcD20* | 36 | 26 | 0.944 | 0.940 | -0.005 | -0.002 |
|  | *PcD52* | 36 | 8 | 0.806 | 0.799 | -0.008 | -0.003 |
|  | *Pc83* | 36 | 13 | 0.833 | 0.848 | 0.017 | 0.008 |
|  | *PcD26* | 36 | 11 | 0.889 | 0.884 | -0.006 | -0.003 |
|  | *PcD53* | 36 | 9 | 0.500 | 0.502 | 0.003 | 0.001 |
| Matador WMA | *Pc41* | 55 | 10 | 0.873 | 0.849 | -0.028 | -0.013 |
|  | *PcD01* | 55 | 18 | 0.891 | 0.905 | 0.016 | 0.008 |
|  | *PcD09* | 55 | 9 | 0.855 | 0.846 | -0.010 | -0.004 |
|  | *Pc70* | 55 | 8 | 0.873 | 0.830 | -0.051 | -0.023 |
|  | *PcD14* | 55 | 14 | 0.909 | 0.885 | -0.027 | -0.013 |
|  | *PcD20* | 55 | 29 | 0.927 | 0.930 | 0.003 | 0.002 |
|  | *PcD52* | 55 | 9 | 0.818 | 0.810 | -0.010 | -0.005 |
|  | *Pc83* | 55 | 20 | 0.891 | 0.897 | 0.007 | 0.003 |
|  | *PcD26* | 55 | 13 | 0.855 | 0.865 | 0.012 | 0.006 |
|  | *PcD53* | 55 | 15 | 0.691 | 0.688 | -0.004 | -0.002 |
| RPQRR | *Pc41* | 78 | 12 | 0.833 | 0.838 | 0.005 | 0.002 |
|  | *PcD01* | 79 | 20 | 0.886 | 0.890 | 0.004 | 0.002 |
|  | *PcD09* | 79 | 13 | 0.810 | 0.878 | 0.077 | 0.036 |
|  | *Pc70* | 79 | 9 | 0.886 | 0.822 | -0.078 | -0.035 |
|  | *PcD14* | 79 | 15 | 0.899 | 0.865 | -0.039 | -0.018 |
|  | *PcD20* | 79 | 31 | 0.949 | 0.941 | -0.008 | -0.004 |
|  | *PcD52* | 79 | 6 | 0.772 | 0.763 | -0.012 | -0.005 |
|  | *Pc83* | 79 | 20 | 0.924 | 0.905 | -0.021 | -0.010 |
|  | *PcD26* | 79 | 14 | 0.924 | 0.873 | -0.059 | -0.027 |
|  | *PcD53* | 79 | 15 | 0.722 | 0.651 | -0.108 | -0.043 |
| CMA | *Pc41* | 20 | 11 | 1.000 | 0.878 | -0.140 | -0.065 |
|  | *PcD01* | 20 | 12 | 0.650 | 0.879 | 0.260 | **0.122*** |
|  | *PcD09* | 20 | 11 | 0.850 | 0.840 | -0.012 | -0.005 |
|  | *Pc70* | 20 | 8 | 0.900 | 0.826 | -0.089 | -0.040 |
|  | *PcD14* | 20 | 10 | 1.000 | 0.869 | -0.151 | -0.070 |
|  | *PcD20* | 20 | 21 | 0.900 | 0.928 | 0.030 | 0.014 |
|  | *PcD52* | 20 | 7 | 0.800 | 0.809 | 0.011 | 0.005 |
|  | *Pc83* | 20 | 15 | 0.750 | 0.833 | 0.099 | 0.045 |
|  | *PcD26* | 20 | 11 | 0.900 | 0.853 | -0.056 | -0.026 |
|  | *PcD53* | 20 | 7 | 0.400 | 0.386 | -0.036 | -0.010 |
| E. New Mexico | *Pc41* | 18 | 11 | 0.944 | 0.836 | -0.129 | -0.059 |
|  | *PcD01* | 18 | 15 | 1.000 | 0.907 | -0.102 | -0.049 |
|  | *PcD09* | 18 | 9 | 0.944 | 0.816 | -0.157 | -0.071 |
|  | *Pc70* | 18 | 9 | 0.833 | 0.793 | -0.051 | -0.022 |
|  | *PcD14* | 18 | 10 | 0.889 | 0.863 | -0.030 | -0.014 |
|  | *PcD20* | 18 | 20 | 1.000 | 0.938 | -0.066 | -0.032 |
|  | *PcD52* | 18 | 10 | 0.889 | 0.861 | -0.032 | -0.015 |
|  | *Pc83* | 18 | 12 | 0.667 | 0.716 | 0.069 | 0.029 |
|  | *PcD26* | 18 | 11 | 0.944 | 0.900 | -0.050 | -0.024 |
|  | *PcD53* | 18 | 6 | 0.389 | 0.448 | 0.131 | 0.041 |
| Colorado | *Pc41* | 12 | 7 | 0.833 | 0.830 | -0.004 | -0.002 |
|  | *PcD01* | 12 | 14 | 0.833 | 0.917 | 0.091 | 0.044 |
|  | *PcD09* | 12 | 11 | 0.917 | 0.878 | -0.043 | -0.020 |
|  | *Pc70* | 12 | 7 | 0.833 | 0.792 | -0.053 | -0.023 |
|  | *PcD14* | 12 | 11 | 1.000 | 0.875 | -0.143 | -0.067 |
|  | *PcD20* | 12 | 13 | 0.917 | 0.906 | -0.011 | -0.006 |
|  | *PcD52* | 12 | 8 | 1.000 | 0.826 | -0.210 | -0.095 |
|  | *Pc83* | 12 | 14 | 0.833 | 0.910 | 0.084 | 0.040 |
|  | *PcD26* | 12 | 9 | 0.750 | 0.816 | 0.081 | 0.036 |
|  | *PcD53* | 12 | 10 | 0.750 | 0.806 | 0.069 | 0.031 |
| Camp Bowie | *Pc41* | 11 | 8 | 0.818 | 0.756 | -0.082 | -0.035 |
|  | *PcD01* | 11 | 8 | 0.909 | 0.851 | -0.068 | -0.031 |
|  | *PcD09* | 11 | 6 | 0.818 | 0.802 | -0.021 | -0.009 |
|  | *Pc70* | 11 | 7 | 0.909 | 0.831 | -0.095 | -0.043 |
|  | *PcD14* | 11 | 11 | 1.000 | 0.893 | -0.120 | -0.057 |
|  | *PcD20* | 11 | 8 | 0.818 | 0.806 | -0.015 | -0.007 |
|  | *PcD52* | 11 | 6 | 0.909 | 0.802 | -0.134 | -0.060 |
|  | *Pc83* | 11 | 11 | 0.909 | 0.880 | -0.033 | -0.015 |
|  | *PcD26* | 11 | 7 | 0.909 | 0.769 | -0.183 | -0.079 |
|  | *PcD53* | 11 | 8 | 0.545 | 0.740 | 0.263 | 0.112 |
| Grey Co. | *Pc41* | 11 | 10 | 0.727 | 0.888 | 0.181 | 0.085 |
|  | *PcD01* | 11 | 10 | 0.909 | 0.884 | -0.028 | -0.013 |
|  | *PcD09* | 11 | 9 | 1.000 | 0.855 | -0.169 | -0.078 |
|  | *Pc70* | 11 | 6 | 0.909 | 0.744 | -0.222 | -0.095 |
|  | *PcD14* | 11 | 8 | 0.909 | 0.798 | -0.140 | -0.062 |
|  | *PcD20* | 11 | 17 | 0.909 | 0.934 | 0.027 | 0.013 |
|  | *PcD52* | 11 | 7 | 0.909 | 0.831 | -0.095 | -0.043 |
|  | *Pc83* | 11 | 10 | 0.727 | 0.876 | 0.170 | 0.079 |
|  | *PcD26* | 11 | 9 | 1.000 | 0.855 | -0.169 | -0.078 |
|  | *PcD53* | 11 | 8 | 0.545 | 0.574 | 0.050 | 0.018 |
| Mitchell Co. | *Pc41* | 14 | 8 | 0.929 | 0.832 | -0.117 | -0.053 |
|  | *PcD01* | 14 | 12 | 0.857 | 0.875 | 0.020 | 0.010 |
|  | *PcD09* | 13 | 6 | 0.769 | 0.811 | 0.051 | 0.023 |
|  | *Pc70* | 14 | 8 | 0.786 | 0.821 | 0.043 | 0.020 |
|  | *PcD14* | 14 | 9 | 0.786 | 0.839 | 0.064 | 0.029 |
|  | *PcD20* | 14 | 20 | 1.000 | 0.939 | -0.065 | -0.032 |
|  | *PcD52* | 14 | 7 | 0.714 | 0.755 | 0.054 | 0.023 |
|  | *Pc83* | 13 | 8 | 0.846 | 0.799 | -0.059 | -0.026 |
|  | *PcD26* | 14 | 9 | 0.786 | 0.849 | 0.075 | 0.035 |
|  | *PcD53* | 14 | 7 | 0.857 | 0.732 | -0.171 | -0.072 |
| Chaparral WMA | *Pc41* | 63 | 14 | 0.841 | 0.854 | 0.015 | 0.007 |
|  | *PcD01* | 63 | 14 | 0.857 | 0.841 | -0.019 | -0.009 |
|  | *PcD09* | 63 | 13 | 0.873 | 0.866 | -0.008 | -0.004 |
|  | *Pc70* | 63 | 11 | 0.794 | 0.847 | 0.063 | 0.029 |
|  | *PcD14* | 63 | 10 | 0.730 | 0.871 | 0.162 | **0.075*** |
|  | *PcD20* | 63 | 23 | 0.889 | 0.930 | 0.044 | 0.021 |
|  | *PcD52* | 63 | 8 | 0.810 | 0.805 | -0.005 | -0.002 |
|  | *Pc83* | 63 | 21 | 0.825 | 0.891 | 0.073 | 0.035 |
|  | *PcD26* | 63 | 13 | 0.825 | 0.852 | 0.031 | 0.014 |
|  | *PcD53* | 63 | 18 | 0.873 | 0.908 | 0.039 | 0.019 |
| Matagorda Island WMA | *Pc41* | 30 | 6 | 0.833 | 0.781 | -0.067 | -0.029 |
|  | *PcD01* | 30 | 10 | 0.600 | 0.851 | 0.295 | **0.136*** |
|  | *PcD09* | 30 | 6 | 0.367 | 0.749 | 0.511 | **0.219**** |
|  | *Pc70* | 30 | 7 | 0.633 | 0.698 | 0.092 | 0.038 |
|  | *PcD14* | 30 | 9 | 0.767 | 0.822 | 0.067 | 0.030 |
|  | *PcD20* | 30 | 16 | 0.767 | 0.877 | 0.125 | 0.059 |
|  | *PcD52* | 30 | 4 | 0.633 | 0.685 | 0.075 | 0.031 |
|  | *Pc83* | 29 | 12 | 0.793 | 0.834 | 0.049 | 0.022 |
|  | *PcD26* | 30 | 7 | 0.800 | 0.765 | -0.046 | -0.020 |
|  | *PcD53* | 30 | 12 | 0.833 | 0.849 | 0.019 | 0.009 |
| Starr Co. | *Pc41* | 10 | 7 | 0.800 | 0.765 | -0.046 | -0.020 |
|  | *PcD01* | 10 | 8 | 0.800 | 0.810 | 0.012 | 0.006 |
|  | *PcD09* | 10 | 8 | 0.800 | 0.785 | -0.019 | -0.008 |
|  | *Pc70* | 10 | 8 | 0.600 | 0.815 | 0.264 | 0.119 |
|  | *PcD14* | 9 | 8 | 0.889 | 0.833 | -0.067 | -0.030 |
|  | *PcD20* | 10 | 7 | 0.800 | 0.820 | 0.024 | 0.011 |
|  | *PcD52* | 10 | 4 | 0.800 | 0.645 | -0.240 | -0.094 |
|  | *Pc83* | 10 | 12 | 0.900 | 0.900 | 0.000 | 0 |
|  | *PcD26* | 10 | 8 | 0.900 | 0.805 | -0.118 | -0.053 |
|  | *PcD53* | 10 | 11 | 0.900 | 0.885 | -0.017 | -0.008 |
