# Supplementary material for: Population genetic structure of Texas horned lizards: implications for reintroduction and captive breeding
Source: PeerJ. 2019 Oct 1;7:e7746. doi: 10.7717/peerj.7746 (PMC6777493; doi:10.7717/peerj.7746)
Supplement: Table S3 — For sampling site names, SP = state park, WMA = wildlife management area, CMA = Cross Bar Management Area, Co. = county, RPQRR = Rolling Plains Quail Research Ranch. [file peerj-07-7746-s003.docx]

|  | Brewster Co. | Hueco Tanks SP | Seminole Canyon SP | Midland Co. | Yoakum Dunes WMA | Matador WMA | RPQRR | CMA | E. New Mexico | Colorado | Camp Bowie | Grey Co. | Mitchell Co. | Chaparral WMA | Matagorda Island WMA | Starr Co. |
| --- | --- | --- | --- | --- | --- | --- | --- | --- | --- | --- | --- | --- | --- | --- | --- | --- |
| Brewster Co. | 0.000 |  |  |  |  |  |  |  |  |  |  |  |  |  |  |  |
| Hueco Tanks SP | 0.066 | 0.000 |  |  |  |  |  |  |  |  |  |  |  |  |  |  |
| Seminole Canyon SP | 0.059 | 0.106 | 0.000 |  |  |  |  |  |  |  |  |  |  |  |  |  |
| Midland Co. | 0.041 | 0.099 | 0.025 | 0.000 |  |  |  |  |  |  |  |  |  |  |  |  |
| Yoakum Dunes WMA | 0.051 | 0.111 | 0.024 | 0.002 | 0.000 |  |  |  |  |  |  |  |  |  |  |  |
| Matador WMA | 0.054 | 0.090 | 0.032 | 0.010 | 0.014 | 0.000 |  |  |  |  |  |  |  |  |  |  |
| RPQRR | 0.062 | 0.104 | 0.028 | 0.006 | 0.013 | 0.009 | 0.000 |  |  |  |  |  |  |  |  |  |
| CMA | 0.050 | 0.115 | 0.029 | 0.004 | 0.006 | 0.016 | 0.017 | 0.000 |  |  |  |  |  |  |  |  |
| E. New Mexico | 0.045 | 0.107 | 0.041 | 0.007 | 0.003 | 0.018 | 0.021 | 0.002 | 0.000 |  |  |  |  |  |  |  |
| Colorado | 0.053 | 0.086 | 0.032 | 0.025 | 0.030 | 0.034 | 0.037 | 0.031 | 0.031 | 0.000 |  |  |  |  |  |  |
| Camp Bowie | 0.069 | 0.098 | 0.047 | 0.038 | 0.040 | 0.027 | 0.035 | 0.031 | 0.048 | 0.030 | 0.000 |  |  |  |  |  |
| Grey Co. | 0.051 | 0.103 | 0.017 | 0.002 | 0.008 | 0.010 | 0.009 | 0.010 | 0.019 | 0.012 | 0.027 | 0.000 |  |  |  |  |
| Mitchell Co. | 0.043 | 0.095 | 0.031 | 0.007 | 0.009 | 0.011 | 0.017 | 0.012 | 0.010 | 0.028 | 0.028 | 0.020 | 0.000 |  |  |  |
| Chaparral WMA | 0.083 | 0.092 | 0.046 | 0.050 | 0.055 | 0.041 | 0.043 | 0.064 | 0.064 | 0.052 | 0.058 | 0.041 | 0.047 | 0.000 |  |  |
| Matagorda Island WMA | 0.114 | 0.110 | 0.101 | 0.090 | 0.092 | 0.079 | 0.082 | 0.102 | 0.103 | 0.080 | 0.088 | 0.080 | 0.081 | 0.061 | 0.000 |  |
| Starr Co. | 0.081 | 0.077 | 0.065 | 0.061 | 0.073 | 0.056 | 0.058 | 0.088 | 0.077 | 0.068 | 0.085 | 0.060 | 0.057 | 0.019 | 0.074 | 0.000 |
