# Supplementary figures and images for: Population genetic structure of Texas horned lizards: implications for reintroduction and captive breeding
Source: PeerJ. 2019 Oct 1;7:e7746. doi: 10.7717/peerj.7746 (PMC6777493; doi:10.7717/peerj.7746)

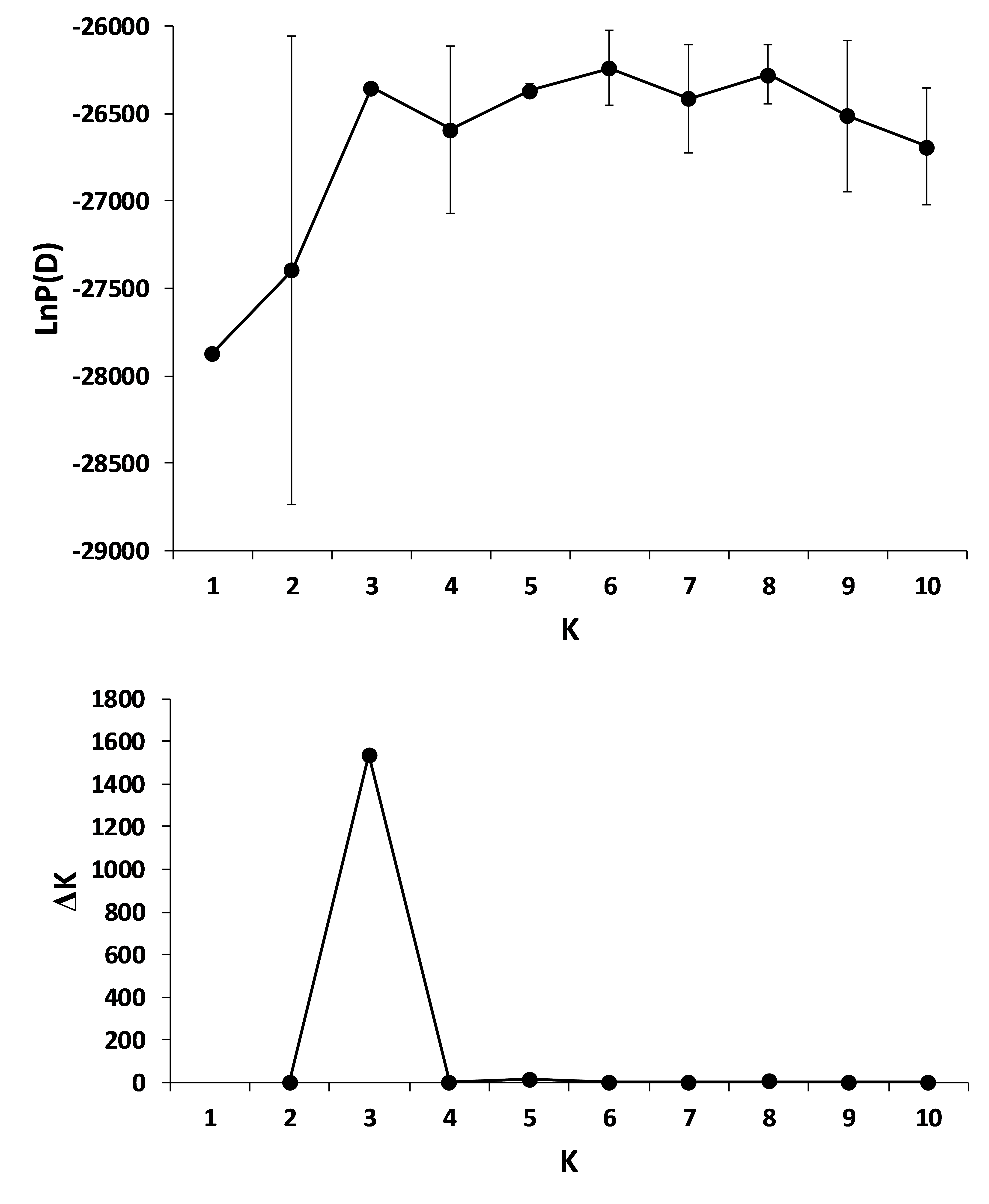

Supplement: Figure S1 — Top panel—mean (±SD) LnP (D) (N = 10 runs) for K clusters, and bottom panel—ΔK for Kclusters (using the method of Evanno, Regnaut & Goudet, 2005) based on 542 Texas horned lizards, Phrynosoma cornutum, sampledfrom across the state of Texas and surrounding areas. [file peerj-07-7746-s004.png]

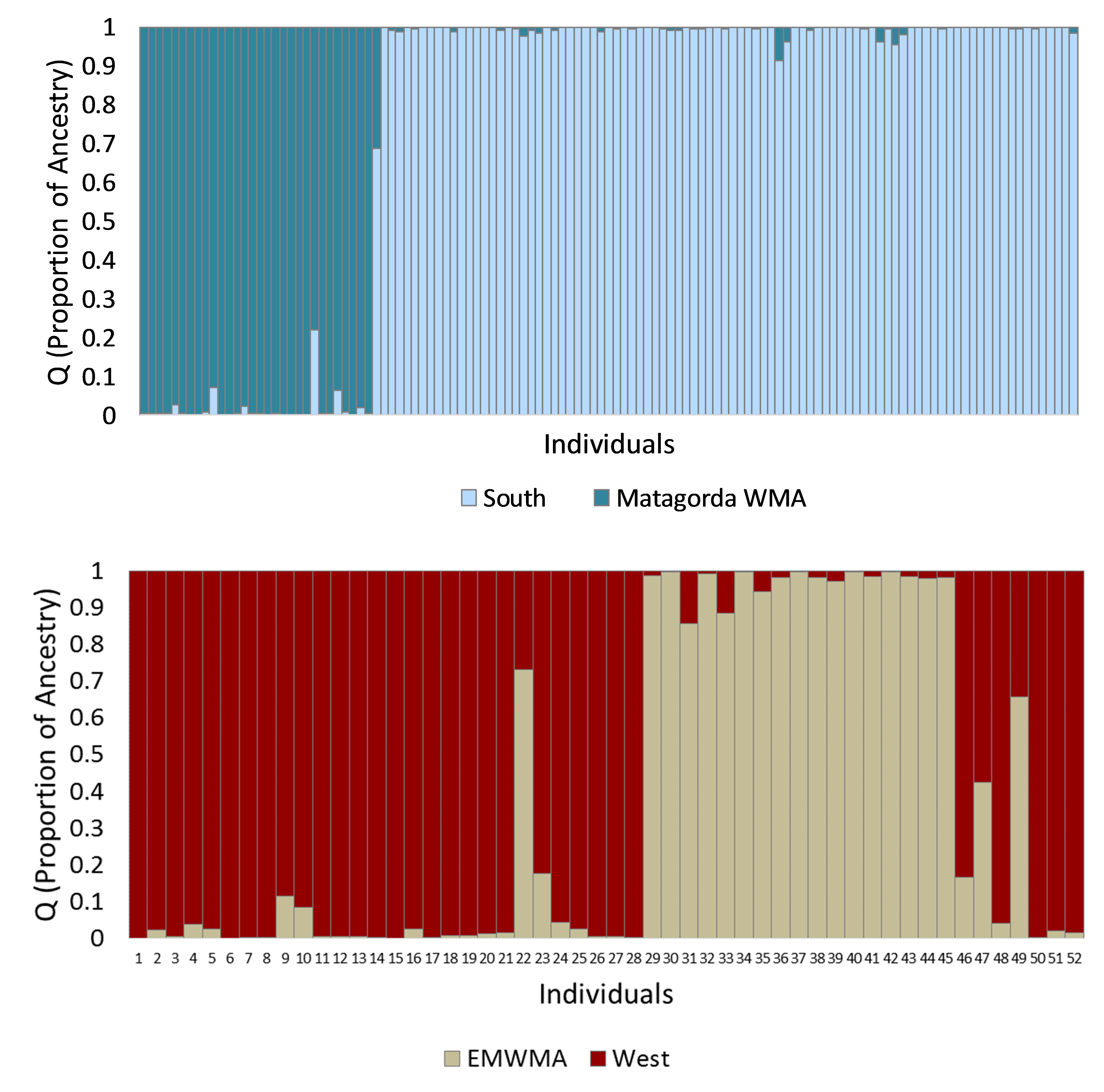

Supplement: Figure S2 — Each vertical bar in the two panels indicates the proportion of ancestry (q) for an individual lizard with the colors representing the cluster or population identified in STRUCTURE. Individual lizards in the south top panel are organized by geographic sampling location, starting with the Matagorada Island WMA individuals and then the mainland. Individual lizards in the west bottom panel are organized by longitude. The tan bars are predominately in individuals from west Texas (individuals 9–49) and especially individuals 29–49 from Brewster Co. near the Elephant Mountain WMA (EMWMA). Individuals 50–52 were found in Colorado and Seminole Canyon SP. [file peerj-07-7746-s005.png]
